# Anatomical, Clinical and Electrical Observations in Piriformis Syndrome

**DOI:** 10.1186/1749-799X-5-3

**Published:** 2010-01-21

**Authors:** Roger M Jawish, Hani A Assoum, Chaker F Khamis

**Affiliations:** 1 Medical School, St Joseph University, Beirut, Lebanon; 2 Department of Orthopaedic, Sacré Coeur Hospital, BP 116 Hazmieh, Lebanon; 3 Department of Electrodiagnostic, Sacré Coeur Hospital, BP 116 Hazmieh, Lebanon

## Abstract

**Background:**

We provided clinical and electrical descriptions of the piriformis syndrome, contributing to better understanding of the pathogenesis and further diagnostic criteria.

**Methods:**

Between 3550 patients complaining of sciatica, we concluded 26 cases of piriformis syndrome, 15 females, 11 males, mean age 35.37 year-old. We operated 9 patients, 2 to 19 years after the onset of symptoms, 5 had piriformis steroids injection. A dorsolumbar MRI were performed in all cases and a pelvic MRI in 7 patients. The electro-diagnostic test was performed in 13 cases, between them the H reflex of the peroneal nerve was tested 7 times.

**Results:**

After a followup 1 to 11 years, for the 17 non operated patients, 3 patients responded to conservative treatment. 6 of the operated had an excellent result, 2 residual minor pain and one failed. 3 new anatomical observations were described with atypical compression of the sciatic nerve by the piriformis muscle.

**Conclusion:**

While the H reflex test of the tibial nerve did not give common satisfaction in the literature for diagnosis, the H reflex of the peroneal nerve should be given more importance, because it demonstrated in our study more specific sign, with six clinical criteria it contributed to improve the method of diagnosis. The cause of this particular syndrome does not only depend on the relation sciatic nerve-piriformis muscle, but the environmental conditions should be considered with the series of the anatomical anomalies to explain the real cause of this pain.

## Background

Since many years, we had a particular interest for the intractable sciatica with failure of long term treatment of lumbar pain. In such cases, our investigation was focused on a suspected piriformis syndrome missing from many decades specific signs for diagnosis.

Yeoman [1] 1928, reported that the sciatica may be caused by a periarthritis involving the anterior sacroiliac ligament, the piriformis muscle and the adjacent branches of the sciatic nerve. Freiberg and Vinke [2] 1934, considered that the inflammation of the sacroiliac joint may primarily cause reaction of the piriformis muscle and its fascia, and secondarly, the irritation of the overlying lumbosacral plexus.

Based on cadaver dissections, Beaton and Anson [3] 1938, gave the hypothesis that the spasm of the piriformis muscle could be responsible for the irritation of the nerve. Robinson [4] 1947, has introduced the term "piriformis syndrome" and applied it to sciatica related to abnormal muscle, which is usually traumatic in origin, with emphasis on the necessity to rule out all other causes of sciatica.

Even though it is commonly accepted that no consensus was defined about the clinical and the laboratory studies, we have tried to describe further clinical criteria that we concluded from the physical examination of patients complaining of sciatica. The electro-diagnostic test is also considered as an important method of diagnosis, while testing of the sciatic nerve has contributed in many studies [5-7] to expect the presence of a piriformis impingement, with a particular interest for the H-reflex of the tibial nerve [7]. We, however, believe that more importance should be given to the H-reflex of the peroneal nerve which has demonstrated more specific signs in our study.

The lack of reliable objective test to identify the piriformis muscle syndrome leads in many cases to great expenses in repetitive imaging studies and to time loss in searching for the origin of the intractable sciatica among the lumbar pathologies. Our clinical criteria concluded from the epidemiologic study and anatomical observations, added to the electrical testing of the peroneal nerve, could improve the method of diagnosis and avoid the delays in unnecessary suffering.

## Materials and methods

Between 1997 and 2007, about 3550 patients complaining of low back pain and sciatica were examined by the first author and not referred by any other physician. We retained 26 cases of piriformis syndrome, 15 women and 11 men, aged between 15 and 66 years (average: 35.37), 14 left and 12 right. 9 patients have accepted the surgery after either, failure of conservative treatment or presence of neuro-muscular deficiencies.

The 17 non operated patients were 10 women and 7 men, aged between 18 and 66, 10 left and 7 right, none had a previous history of trauma to the gluteal region; 4 were athletics (one gymnastics, 2 walkers and one basketballer). The time average from the beginning of the pain to the treatment was 3.14 years (range: 1 month to 11 years). One patient had a failed previous lumbar disc surgery for sciatica. Five of them have benefited from intrapiriformis muscle steroids injection.

The 9 operated patients (table 1) were 5 women and 4 men, aged between 15 and 65 (average: 35.88), 4 left and 5 right. The weight average was 73.88 Kg (range: 55 to 110). Six athletics distributed between 3 walkers, 2 footballers and 1 swimmer, only one patient had a previous history of a fall onto a buttock, 3 months before the onset of the symptoms. All patients had followed a preoperative medical treatment including painkillers and muscle relaxants; three have also had intrapiriformis muscle steroids injection. The time average from the beginning of the pain to surgery was: 5.44 years (range, 2 to 19 years).

**Table 1:** Clinical Data on 9 operated patients

Patient	1	2	3	4	5	6	7	8	9
Sex	M	f	m	f	f	m	m	f	f

Age(years)	32	32	58	23	44	15	39	15	65

Weight(kg)	70	60	99	58	57	110	76	55	80

Side	L	L	L	R	L	R	R	R	R

Sport	-	-	Football	Walker	Swim	football	Walker	Walker	-

Gluteal trauma	-	-	-	-	-	yes	-	-	-

Preop. Steroid injection	0	1	3	0	2	0	0	0	0

Delay to surgery (years)	3	7	3	4	2	3	6	2	19

Sciatica	yes	yes	yes	Drop foot	yes	yes	yes	yes	yes

Pain on sitting position	+	+	+	+	+	+	+	+	+

Gluteal atrophy	-	-	-	+	-	-	+	+	+

Pain on digital pressure	+	+	+	+	+	+	+	+	+

H-reflex peroneal nerve	+	+	+	+	+		+		+

Preop.MRI (spine)	1	1	4	7	3	1	3	2	1

Preop.MRI (pelvis)	Veinous sign	Piriformis hypertrophy		Veinous sign	Veinous sign	Piriformis hypertrophy	Veinous sign		Normal

From surgery to pain relief	One year	6 months	3 months	2 weeks	No relief	1 year	1 year	4 months	1 month

Residual gluteal pain	-	+	-	-	+	-	-	-	+

Functional result	Excellent	Good	Excellent	Excellent	Bad	Excellent	Excellent	Excellent	good

The preoperative and last followup evaluation concerning the clinical status and the results of the MRI images and the H-reflex of the peroneal nerve.

The neurological preoperative examination showed one complete right drop foot, and one patient was obliged to stand up in a triple flexion position, in prolonged standing; 5 patients had dysesthesia and altered reflexes; 4 patients had gluteal atrophy at the affected side and one patient had posterior leg atrophy.

All patients of the study benefited of a dorsolumbar MRI, none of them has revealed nerve root compression or any spinal pathology responsible of the sciatica. A pelvic MRI has been performed in 7 patients and has demonstrated an obvious hypertrophy of the homolateral piriformis muscle in two cases, and in 4 cases, there were mild congestion of the venous plexus around the sciatic nerve.

**Figure 1:**
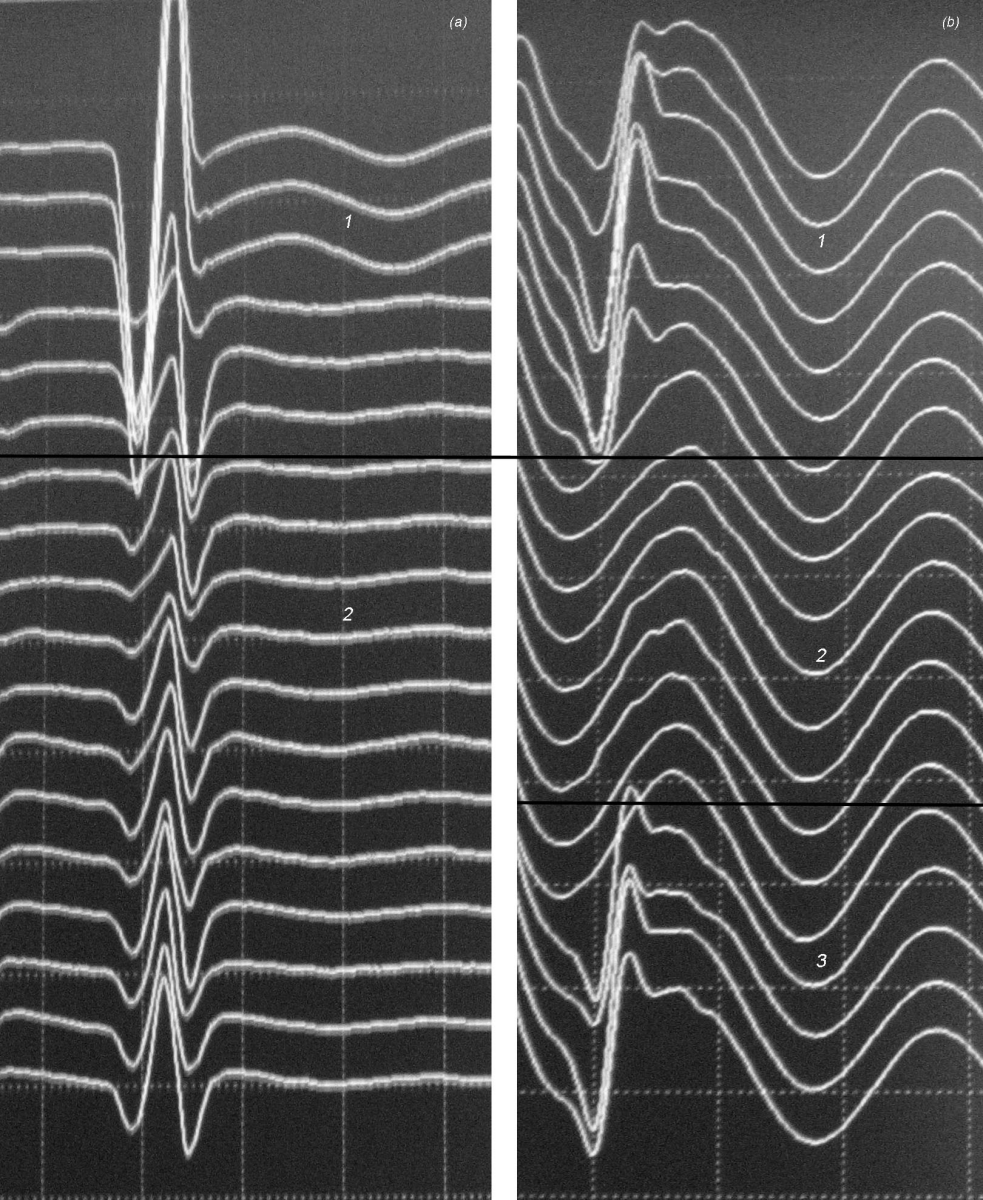
**Electro-diagnostic test of a 22 year-old female patient complaining of right sided piriformis muscle syndrome since 6 years**. (A-1) The H reflex of the tibial nerve, the leg in a straight position, was normal, (A-2) showed slight disturbance of the H wave, during the stress maneuver of flexion and internal rotation of the lower limb. (B-1) the H-reflex of the common peroneal nerve, the leg in a straight position, was normal, (B-2) noted the complete extinction of the H wave, during the painful maneuver of forced adduction-internal rotation, (B-3) the H reflex reappeared when the leg was returned in the relieved straight position.

The EMG was performed on 13 patients. Only three of them have shown alteration of the H reflex of the tibial nerve. For the last seven patients, we started to explore the H reflex of the common peroneal nerve. We observed during the EMG recording, a complete disappearance of the peroneal's H reflex when the affected lower limb was put in the pain position (internal rotation and adduction); the H reflex reappeared when the limb was returned to the relieved straight position (Fig. 1). When this test was performed at the unaffected opposite site, the H reflex remained normal in all positions.

The various tests performed in our series have revealed constancy of the following signs in all our patients: 1)Absence of any spinal pathology at the dorsolumbar MRI. 2) Tenderness with digital pressure of the sciatic spine and absence of pain complaint at the lower back and the sacroiliac joint. 3) Intolerance to sitting on the involved side with the body inclined over the thigh. 4) Sciatica in the sitting position when the homolateral leg is crossed over the unaffected side. 5) Exacerbated sciatica by the maneuver of internal rotation and maximal adduction of the hip. 6) The H reflex tested for the common peroneal nerve (EMG) has disappeared in pain position with internal rotation and forced adduction.

## Results

### Clinical outcome

Considering the 17 none operated patients and after a follow up ranging from one to 11 years, we have obtained the following results: one patient has responded to medical treatment, one was operated by another surgeon for piriformis muscle syndrome with a good result, two have responded to infiltration, seven have not responded to conservative measures and six patients were missed.

After a follow up between 1 and 11 years, the 9 operated patients have been interrogated and reexamined by the senior author and noted a relief of pain in 2 weeks to 12 months after the operation (mean 5.61 months). Six patients have obtained an excellent result with a complete relief of pain even in prolonged periods of sitting. Two patients have reported minor residual pain in the buttock precipitated by strenuous activities. One patient has considered that the operation was not beneficial to her knowing that we were not able to examine her (table 1).

The five patients with preoperative sensory problems have had a transient tinnel sign for a maximum of five months, and one of them has demonstrated a paresthesia in the territory of deep peroneal nerve. The patient with a drop foot has recovered within six months. None of the patients had used walkers or crutches postoperatively. We have observed one postoperative transitory limp and one superficial cutaneous infection.

### Operative findings

**Figure 2:**
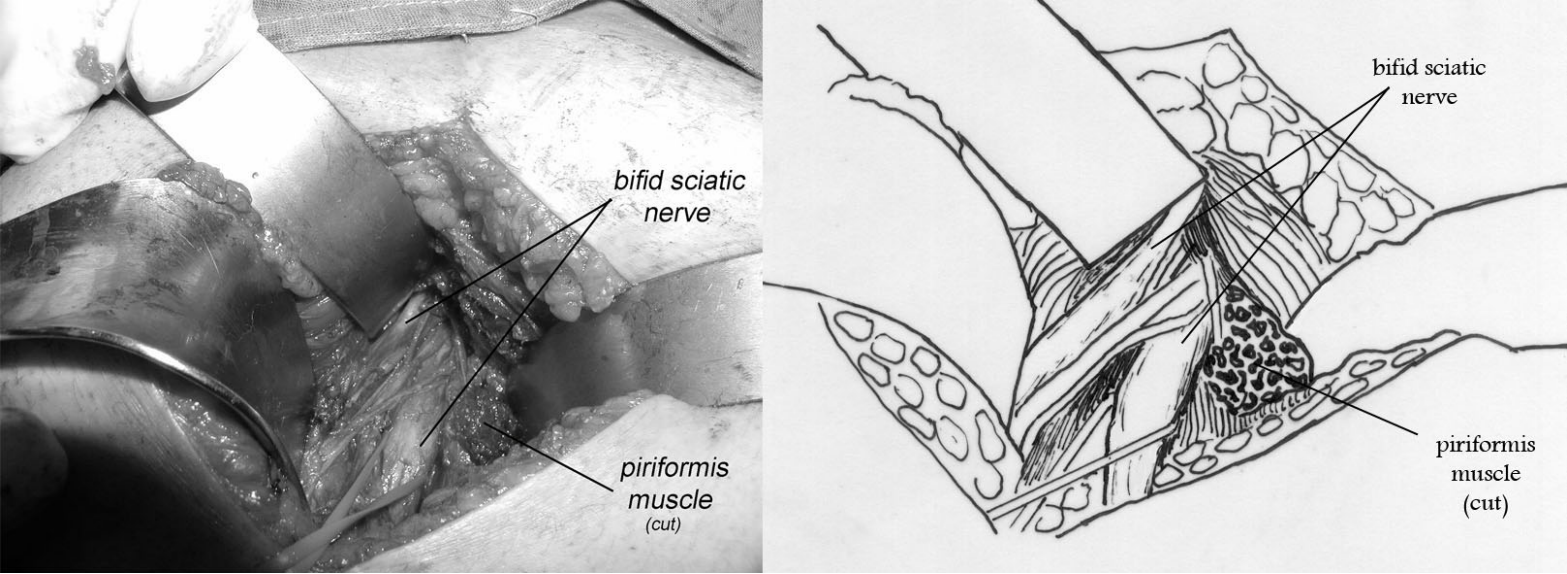
**A 23-year-old female complaining of right sided piriformis muscle syndrome since 4 years**. We noted intraoperatively a bifid sciatic nerve passing under the hypertrophied piriformis muscle.

**Figure 3:**
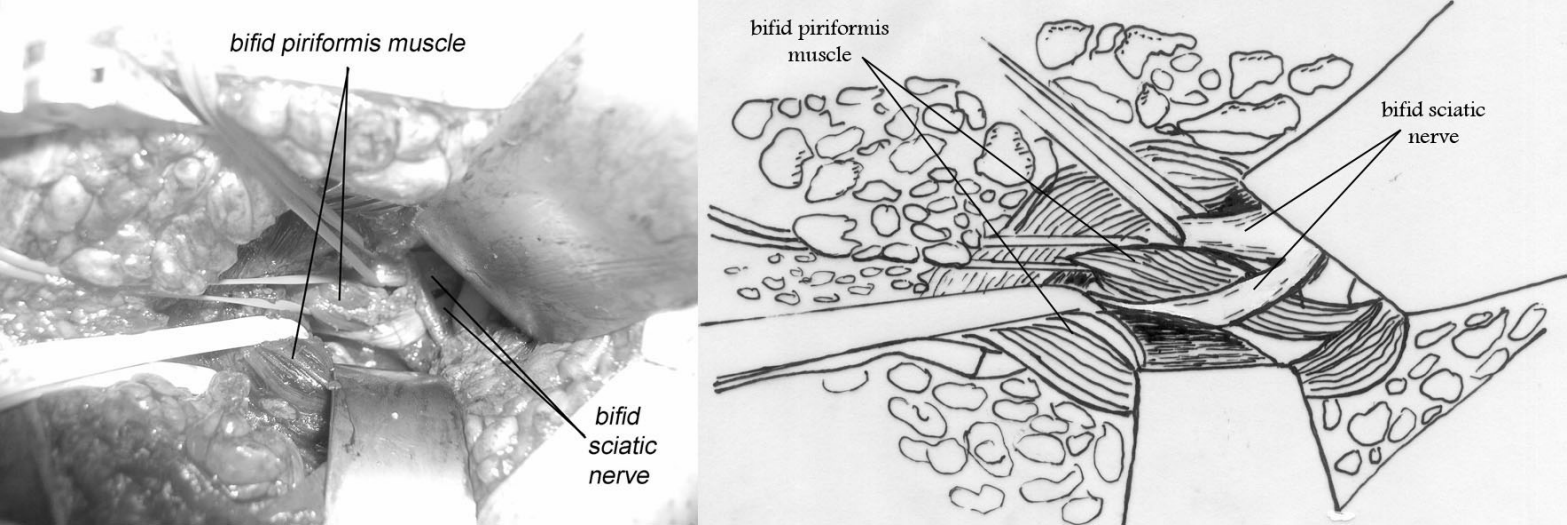
**32-year-old female complaining of left sided piriformis muscle syndrome since 7 years**. We noted intraoperatively a bifid piriformis muscle and a bifid sciatic nerve, one branch of the nerve passing proximal to the muscle and the other one through the split

**Figure 4:**
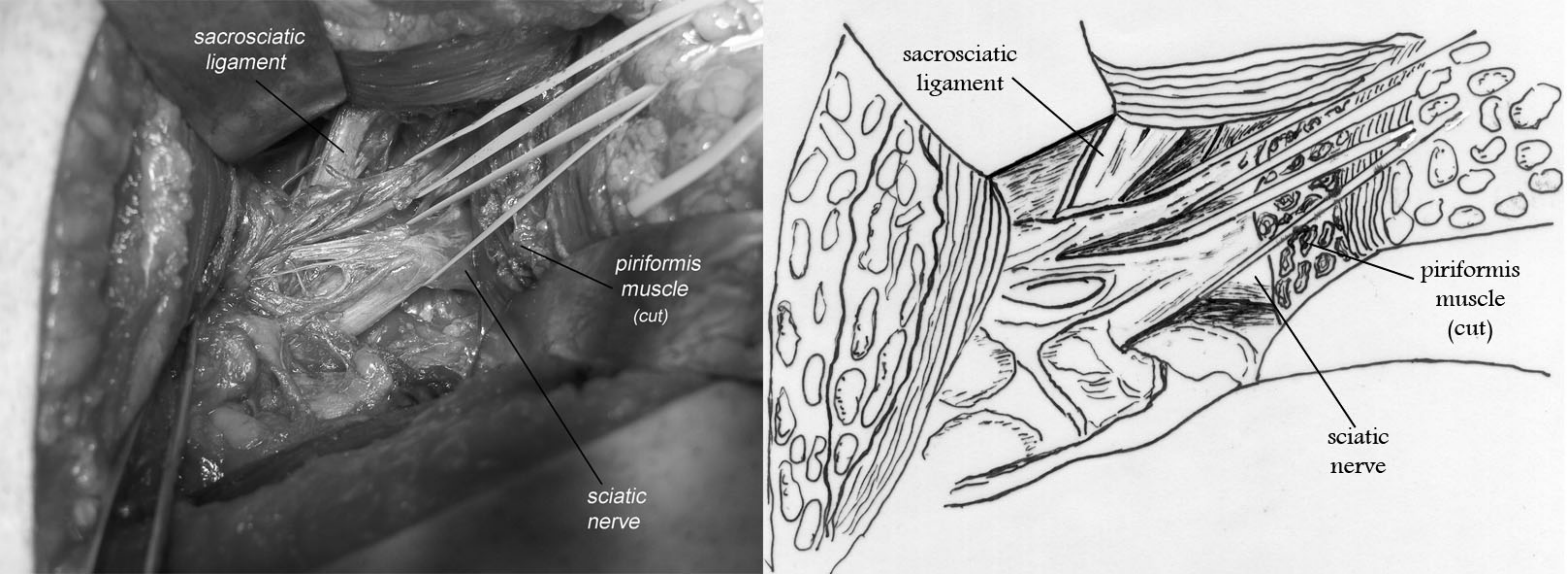
**A 65-year-old female complaining of right sided piriformis muscle syndrome since 19 years**. Note the impingement of the sciatic nerve in contact with the sacrospinous ligament.

**Figure 5:**
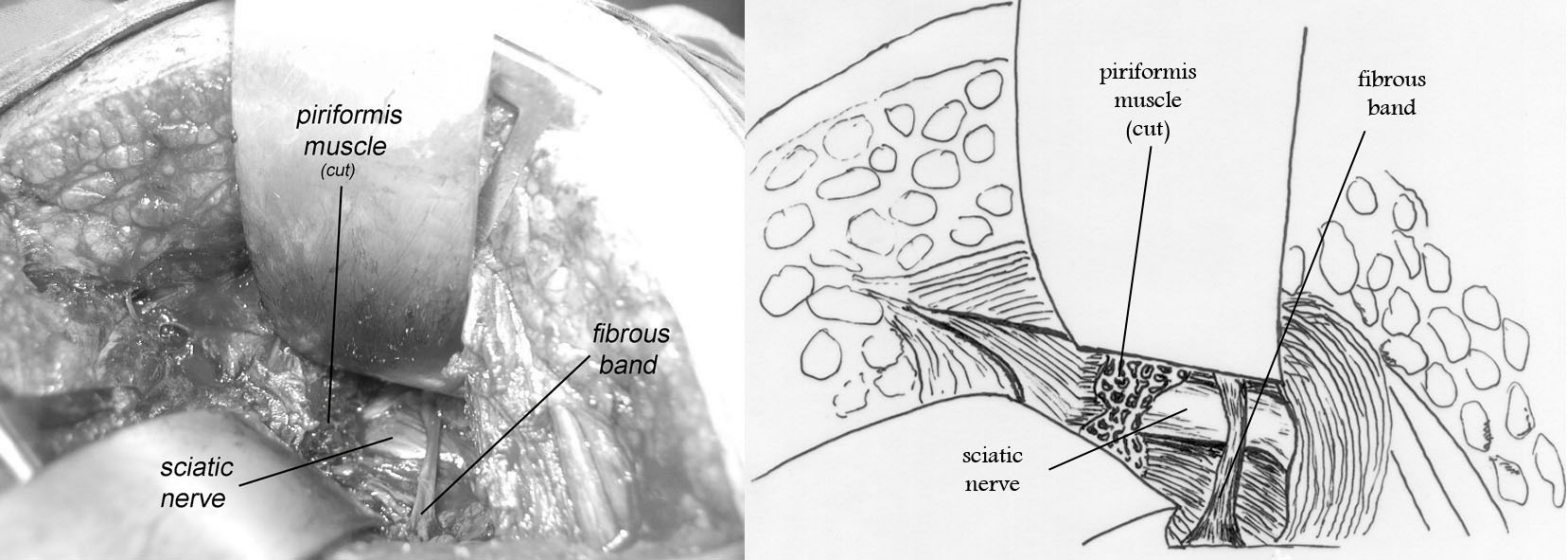
**A 58-year-old male complaining of left sided piriformis muscle syndrome since 3 years**. Note the transverse fibrous band squeezing the sciatic nerve.

In a prone position using Kocher-langenbeck incision, the piriformis muscle was reached through the fibers of the gluteus maximus and sectioned after dissection of the nerve. A neurolysis of the sciatic nerve was performed in all the cases. The intra operative observations of the 9 cases were as following:

The sciatic nerve was bifid passing under the hypertrophied piriformis muscle, 1 case (fig. 2). A bifid piriformis muscle and a bifid sciatic nerve, one branch of the nerve was passing proximal to the muscle and the other one through the split, 1 case (fig. 3). A sciatic impingement by the piriformis muscle and the sacrosciatic ligament, 1 case (fig. 4). The piriformis muscle was hypertrophied, squeezing the sciatic nerve which passed directly below it, 2 cases. A transverse fibrous band compressed the sciatic nerve, 1 case (fig. 5). A nervous connection existed between the sciatic nerve and the inferior gluteal nerve, 1 case. There was no evidence of anatomical impingement of the sciatic nerve in three cases. Congested tortuous veins around the sciatic nerve sight were present in almost all the patients.

## Discussion

It is well known among the authors who studied the piriformis syndrome that many patients treated for low back pain could have sciatic nerve impingement at the buttock. Since the extended use of MRI to evaluate spinal disorders, the piriformis muscle syndrome has become a more separate entity even though the related specific signs were not completely defined and the mechanism is still obscure.

Although the incidence of this affection remains controversial, it was increasing progressively with the improvement of investigations. Most of the reported cases were sporadic, but the latest series described more cases with variable incidence, from 0.33% [8] to 6% [9] depending on the nature of the referral system to the investigators. However, in patients referred for spinal disorders after failure of the treatment, the maximal rate was 5% for Parziale [10] and 14/93 for Benson [5]; although in 1997, Goldner [11] has criticized this high rate and considered that the prevalence in a referral orthopaedic surgery should not exceed 1%, which is close to our value (0.7%) but in a none referral practice.

Regardless of the physiopathologic origin of the complex disorder (muscular or nervous), symptoms and imaging should be combined to confirm the diagnosis. Contrary to many authors [1,2,4], we agree with Bernard and Kirkaldy-Willis [8] that there is no relation between the sacroiliac joint syndrome and the piriformis syndrome, and we also consider that the absence of sacroiliac pain is an essential sign for a positive diagnosis.

Based on two observations, Robinson [4] described the cardinal features of the syndrome with six criteria: (I) a history of trauma to the sacroiliac and gluteal regions; (II) pain in the region of the sacroiliac joint, greater sciatic notch, and piriformis muscle that usually extends down the limb and causes difficulty with walking; (III) acute exacerbation of pain caused by stooping or lifting; (IV) a palpable sausage-shaped mass, tender to palpation, over the piriformis muscle on the affected side; (V) a positive Lasègue sign; and (VI) gluteal atrophy, depending on the duration of the condition.

Many authors [4-6,12,13] have considered trauma in the gluteal area as the major cause of piriformis syndrome, which was not the rule in our series where trauma was evocated in one case only. We, however, believe that piriformis syndrome could be related to exacerbated rotators activity as it was observed in patients with hard physical activity, walkers, athletics and footballer or with repetitive trauma of nerve in patients with prolonged sitting position.

Among all the signs reported in the literature, we have accepted the pain induced by passive internal rotation and adduction of the hip described by Freiberg [2], but the pain induced by resisted abduction and external rotation of the affected thigh, as described by Pace [12], was not in our series a specific sign of this syndrome. However, we have considered pathognomonic the signs which were constantly observed in all the patients of our study, and we have excluded all others that were uncommon as impressive gluteal atrophy, or a palpable sausage-shaped mass [13].

While the cases reported in the past have suffered from none contribution of the modern imaging, the use of MRI has become essential to rule out any spinal disorders or pelvic disorders as mentioned by Pecina [14] who found an MRI abnormality for the piriformis muscle syndrome in 7 out of his 10 patients; it is in practice the first exam that evokes the piriformis muscle, particularly in patient with chronic sciatica. However, and apart from the MR neurography or piriformis blocks [15,16] in which we have no experience, the MRI of pelvis remains unable to define a criteria for diagnosis, since the asymmetrical size of the Piriformis muscle observed in our cases, is common in normal people and identified in T1-weighted MRI of the pelvis performed for 100 persons [17].

The electromyographic is another test for diagnosis, but nerve conduction results reported in the literature were not conclusive and their methods were very controversial. However, it is well admitted that the tibial division of the nerve is usually spared [6] and the inferior gluteal nerve that supplies the gluteus maximus may be affected and the muscle atrophied as observed in four cases of our series. It is well accepted that the impingement of the sciatic nerve should delay the H-reflex as described by Fishman [7], whereas many authors [5,6] have obtained variable results concerning the tibial nerve.

We, however, have demonstrated that the H reflex of the peroneal nerve was more reliable than testing of the tibial nerve, and we have constantly observed extinction of the H wave, during the painful maneuver of forced adduction-internal rotation of the affected leg. In the same condition of stress test, the H reflex of the tibial nerve remained normal for 10 of 13 patients. We believe that fibers of the peroneal nerve could be more vulnerable because they are anatomically more exposed to injury at the buttock in case of trauma or impingement. This electrical testing of peroneal's H-reflex and the clinical criteria constantly observed in all the patients suffering from a nondisk sciatica, could help to prove the diagnosis or reveal more clearly the presence of the entrapment.

The anatomical studies of the piriformis muscle reported in the literature did not contribute to make a real correlation between the clinical signs and the anatomy and to describe the different anatomical forms for the same syndrome. A study [3] involving 240 cadaver dissections has revealed that in 90 percent of cases the sciatic nerve emerges from below the piriformis muscle, in 7 percent the piriformis and the sciatic are divided, one branch of the sciatic nerve passing through the split and the other branch passing distal to the muscle, in 2 percent only the sciatic nerve is divided and in 1 percent the piriformis is divided by the sciatic nerve. Pecina M. found that in 6.15% of cases, the nervous peroneus communis passes between the tendinous parts of m. piriformis, and he considers this variation of practical significance for the development of the Piriformis Syndrome [18]. After reviewing the cadaveric anatomical variants of the literature [3,19] and surgical anatomical descriptions [5,20-22], we demonstrated three anatomical observations in our series (Fig. 2,3,4), but they did not add further information on the anatomical variants and their clinical expressions.

Considering the different anatomical findings, we think that the real cause of this particular syndrome does not only depend on the relation sciatic nerve-piriformis muscle, because the incidence of the anatomical anomalies of these entities is definitely superior to those treated in the reported cases. We, however, lay emphasis on the environmental aspect of this affection, considering the physical activity and lifestyle of the patient which could be an essential factor in revealing an underlying inadaptable anatomy.

## Conclusion

The observations added to those of the literature have contributed to prove the diversity of the anatomical forms of this syndrome which remains very controversial to many surgeons.

We have defined a group of clinical signs, imaging findings and EMG testing which could contribute to avoid diagnostic mistakes and the confusion with the multiple spinal disorders. The environmental conditions should be considered with the anatomical anomalies to explain the real cause of this pain.

## Competing interests

The authors declare that they have no competing interests.

## Authors' contributions

RJ carried out the surgery, defined the different anatomical descriptions and conceived the H-reflex of the peroneal nerve. HA tested the clinical follow-up and helped to draft the manuscript. CK performed the electro-diagnostic test. All authors read and approved the final manuscript.
